# Evolving the l-lysine high-producing strain of *Escherichia coli* using a newly developed high-throughput screening method

**DOI:** 10.1007/s10295-016-1803-1

**Published:** 2016-07-01

**Authors:** Yan Wang, Qinggang Li, Ping Zheng, Yanmei Guo, Lixian Wang, Tongcun Zhang, Jibin Sun, Yanhe Ma

**Affiliations:** 1 College of Biotechnology, Tianjin University of Science and Technology, Tianjin, People’s Republic of China; 2 Key Laboratory of Systems Microbial Biotechnology, Chinese Academy of Sciences, Tianjin, People’s Republic of China; 3 Tianjin Institute of Industrial Biotechnology, Chinese Academy of Sciences, Tianjin, People’s Republic of China

**Keywords:** Lysine production, *Escherichia coli*, Lysine biosensor, High-throughput screening, Strain evolution

## Abstract

This study provided a new method which applied a selected l-lysine-inducible promoter for evolving lysine industrial strains of *E. coli*. According to the intracellular levels of the enhanced green fluorescent protein (EGFP) whose expression was controlled by the promoter, 186 strains were preliminarily selected using fluorescence-activated cell sorting from a 10-million-mutant library generated from a l-lysine high-producing *E. coli* strain. By subsequent multiple parameter evaluation of the 186 selected strains according to the concentration and the yield of lysine, the productivity per unit of cell in 96-deep-well blocks, two mutants MU-1 and MU-2 were obtained. They produced 136.51 ± 1.55 and 133.2 9 ± 1.42 g/L of lysine, respectively, in 5-L jars. Compared with the lysine concentration and the yield of the original strain, those of strain MU-1 improved by 21.00 and 9.05 %, respectively, and those of strain MU-2 improved by 18.14 and 10.41 %, respectively. The mutant selection and evaluation system newly established in our study should be useful for continuous improvement of the current *E. coli* strains in the lysine industry.

## Introduction

l-lysine is one of the nine amino acids essential for human and animal nutrition. It is predominantly used as an additive in animal feed. Its demand has been steadily increasing in recent years, and more than 2.2 million tons of lysine salts are annually produced worldwide by microbial fermentation. As the major industrially using lysine producers, strains of *Escherichia coli* and *Corynebacterium glutamicum* have their respective advantageous properties [1]. Although the potential safety problems of engineered *E. coli* strains are concerned, after purification to avoid the production strain or its components, the lysine product manufactured by fermentation with the *E. coli* strain has been confirmed to be safe [2].

The production strain is the decision-maker for industrial fermentation, largely affecting the economic and environmental performance of a biotechnological process. For several decades, metabolic engineering of lysine-producing *E. coli* strains based on the existing knowledge of the genetic information has been extensively applied [1, 3–7]. To date, the reported best *E. coli* strain produced lysine at a concentration of 134.9 g/L, a yield of 45.4 % (lysine/glucose, W/W) and a productivity of 1.9 g/(L h) [8]. The maximum theoretical lysine yield by the *E. coli* strain is about 68.2 % (lysine/glucose, W/W) [1]. Therefore, the current lysine-producing *E. coli* strains are far from optimal. However, our knowledge on metabolism and regulation of the *E. coli* strain is still incomplete [9, 10], and it is difficult to further accurately optimize the lysine high-producing industrial strains by pure rational metabolic engineering.

In parallel with knowledge-based metabolic engineering, the mutagenesis and the screening method offer another way for strain optimization, less dependent on the existing knowledge. However, the majority of target metabolites do not confer an easily detectable phenotype on the producing cells. Traditionally, the productivity of each genetic variant has to be analyzed with time-consuming, laborious, and expensive analytical methods, such as chromatography and mass spectrometry, leading to low efficiency of the screening process. In nature, the concentrations of chemicals can often be sensed by diverse molecular devices, such as allosteric enzymes, transcriptional factors, and riboswitches. Artificial biosensors developed using such devices can respond to chemical signals and transfer them to easily detectable signals such as fluorescence that can be detected by the fluorescence-activated cell sorter (FACS) [11–15]. A lysine riboswitch from the aspartate kinase III gene (*lysC*) of *E. coli* was used to construct a lysine biosensor. The biosensor was further used to develop a high-throughput screening (HTS) method to evolve a chimeric aspartate kinase and optimize the expression level of phosphoenolpyruvate carboxylase in lysine non-producing *E. coli* strains [16, 17]. The selected strains in each of these two reports produced about 0.75 and 0.67 g/L of lysine in flasks, respectively [16, 17]. A *lysC* riboswitch was reported to bind to l-lysine with an apparent dissociation constant of about 1 μmol/L (about 0.15 mg/L) [18]. The intracellular lysine concentration of wild-type *E. coli* strains was reported to vary from about 361 to 762 μmol/L [19]. The concentration should be higher in lysine high-producing *E. coli* strains. Therefore, the *lysC* riboswitch-based biosensor might not work well therein.

To further improve the existing lysine high-producing *E. coli* strains with the aid of the mutagenesis and HTS method, a biosensor that can respond to a much higher concentration of lysine than the *lysC* riboswitch is desired. In this study, we characterized several previously reported l-lysine-inducible molecular devices [20, 21], and developed a lysine-biosensor practical in lysine high-producing *E. coli* strains. With the aid of the biosensor, an HTS method was constructed and successfully used to improve the strains.

## Materials and methods

### Chemicals and enzymes

l-lysine was supplied by Sinopharm Chemical Reagent Co.,Ltd (Tianjin, China), and 3-morpholinopropanesulfoinc acid (MOPS) was supplied by Amresco (USA). The chemicals *o*-nitrophenol-β-d-galactopyranoside (ONPG) and *o*-nitrophenol were supplied by Solarbio (Beijing, China). Other chemicals used in this study were of analytical grade or better. Restriction endonucleases were purchased from Fermentas (USA). DNA polymerase was obtained from Transgene (Beijing, China). T4 DNA ligase was purchased from New England Biolabs, Inc. (Beijing, China).

### Strains and plasmids

The strains, plasmids, and primers used in this study are listed in Table 1. Other plasmids and strains were constructed based on them.

**Table 1:** Strains, plasmids, and primers used in this study

Strains/plasmids/primers	Description	Source/restriction site
Strains		
MG1655	A substrain of* E. coli* K-12, with a GenBank accession no.: NC_000913.3	Lab stock
LYS1	Derived from an* E. coli* strain DL2 which was constructed in our lab by introducing a recombinant plasmid pTrc99A-dhdps-aspk expressing a DhdpS mutant (E84T) and an LysC mutant (T253R) into MG1655 [22]. LYS1 differed from DL2 in that it expressed another LysC mutant (D340P) [23], and the gene promoter of DhdpS from MG1655 [24] was used to control the expression of DhdpS and LysC separately	Lab stock
LYS2	A mutant of LYS1 with higher lysine productivity	Lab stock
LYS2D	Derived from LYS2 by elimination of its plasmid pTrc99A-dhdps-aspk, without lysine-producing ability	Lab stock
Plasmids		
pET21a-egfp	With an enhanced green fluorescent protein gene (egfp) cloned into the plasmid pET21a	Lab stock
pSB4K5-I52002	Kanamycin resistance, GenBank accession no.: EU496099 [25]	Lab stock
pTrc99A	Ampicillin resistance, GenBank accession no:. U13872	Lab stock
Primers		
BTT-1	AATGAATTCCAGAAGCGGTCTGATAAAACAGAATTTGCC	EcoRI
BTT-2	CAACAGTATGCGCAGCCATAGAAAAATAAACAAAAAGAG	
pN1	CTCTTTTTGTTTATTTTTCTTGCTTAATTTCCTCGGCA	
pN2	CTCCTTCTTAAAGGCGCGCCATAGTGTTTGAAGTTGCCTTT	
pA1	CTCTTTTTGTTTATTTTTCTATGGCTGCGCATACTGTTG	
pA2	CTCCTTCTTAAAGGCGCGCCATAGGGGCACCTACCGAGG	
LacZP-P1	TATGGCGCGCCTTTAAGAAGGAGATATACATATGACCATGATTACGGATTC	AscI
LacZP-G1	TAAGAAGGAGATATACATATGACCATGATTACGGATTCACTGGC	
LacZP-2	GCCACTAGTTTATTTTTGACACCAGACCAACTGGTAATGGTAGCG	SpeI
Egfp-1	TATGGCGCGCCTTTAAGAAGGAGATATACATATGGTGAGCAAGGGCGAGG	AscI
Egfp-2	GCCACTAGTTTACTTGTACAGCTCGTCCATGCCGAGAGTGATCCCG	SpeI
LysGE-1	CTCGAATTCCTAAGGCCGCAATCCCTCGATTGCTGCATCAACG	EcoRI
LysGE-2	GGTCATATGTATATCTCCTTCTTAAAGTCATCTAGGTCCGATGGACAGTAAAAGACTGG	

For construction of recombinant strains, the target nucleotides were obtained and linked to corresponding plasmids which were then used to transform target strains. The terminators *rrnB* T1 and *rrnB* T2 (*rrnB* T1T2) in the plasmid pTrc99A were cloned by PCR with a pair of primers BTT-1 and BTT-2 (Table 1). The gene promoter of NADPH-dependent glutamate synthase beta chain and related oxidoreductases [20] named pN in this study was amplified by PCR using the genome of *C. glutamicum* strain 13032 as a template with a pair of primers pN-1 and pN-2 (Table 1). The putative gene promoter of anthranilate synthase component I [20] named pA was amplified like pN with a pair of primers pA-1 and pA-2 (Table 1). The 13032 genomic region encompassing the regulatory protein LysG gene and the gene promoter of *lysE* under the regulation of LysG was amplified using primers LysGE-1 and LysGE-2, and was named lysGE [21]. The beta-D-galactosidase gene (*lacZ*) of *E. coli* MG1655 was amplified with primers LacZP-P1 and LacZP-2 (PCR product named *lacZ*-*P*), or primers LacZP-G1 and LacZP-2 (PCR product named *lacZ*-*G*). Then, fusion PCR was carried out with BTT-1 and LacZP-2 as primers to fuse *rrnB* T1-T2, pN and *lacZ*-*P* together, and to fuse *rrnB* T1T2, pA and *lacZ*-*P* together. Primers LysGE-1 and LacZP-2 were used to fuse *lacZ*-*G* and *lysGE* together. The fusion PCR products were digested with *Eco*RI and *Spe*I, and ligated separately to the plasmid pSB4K5-I52002 digested with the same restriction enzymes. The generated plasmids pSB4K5-*rrnB*T1T2-pN-*lacZ*, pSB4K5-*rrnB*T1T2-pA-*lacZ,* and pSB4K5-lysGE-*lacZ* were named pNZ, pAZ, and pGZ, respectively. The *egfp* gene was amplified from the plasmid pET21a-*egfp* with a pair of primers EgfpP-1 and EgfpP-2 (Table 1). The PCR product was digested with *Asc*I and *Spe*I, and ligated to the plasmid pAZ digested with the same restriction enzymes. The generated plasmid pSB4K5-*rrnB*T1T2-pA-*gfp* was named pAG. The constructed plasmids were used to transform corresponding strains to generate LYS2D (pNZ), LYS2D (pAZ), LYS2D (pGZ), MG1655 (pAG, pTrc99A), LYS1 (pAG), and LYS2 (pAG).

### Cultivation conditions

For routine cultivation, Luria–Bertani (LB) medium was used. For fermentation, a previously reported fermentation medium [22] was used. When fermentation was carried out in flasks or 96-deep-well plates (Corning Costar 3960 square V-bottom, 2 mL), MOPS was supplemented at a final concentration of 0.4 mol/L to buffer the pH. According to the resistance of cultivated strains, kanamycin was used at a final concentration of 25 mg/L, and/or ampicillin was at 100 mg/L.

All of the cultivation and fermentation processes were performed at 37 °C. Cultivation with flasks was performed in 500 mL flasks containing 20 mL culture, shaking at 220 rpm. Fermentation in 96-deep-well plates was performed with 300 μL medium in each well in a Microtron incubator (Infors) shaking at 850 rpm. Fermentation in 5-L jar fermenters (Shanghai BaoXing Bio-engineering Equipment Co., Ltd, China) was carried out in two precultures and a main fermentation procedure modified from a method in a former report [22]. The first and second precultures were performed with LB medium and the fermentation medium (with corn syrup at 50 g/L instead of 25 g/L), respectively. After the culture reached an OD_600_ of about 5, 250 mL of the second preculture was transferred to the 5-L fermenter containing 1750 mL of the fermentation medium to initiate the fed-batch fermentation process. The pH of the culture was maintained at 7.0 with 25–28 % ammonia water. The aeration rate was 1 VVM (volume of air per volume of medium per minute). The stirring was kept at 600 rpm for the first 6 h and adjusted to 850 rpm, thereafter. Glucose solution at 500 g/L was continuously supplied to maintain the concentration of glucose at 5–10 g/L. Ammonium sulfate solution at 500 g/L was continuously supplied to maintain the concentration of ammonia–nitrogen at 0.05–0.1 g/L.

### Measurement of the specific activity of LacZ

The cultured cells were washed and resuspended in 100 mmol/L potassium phosphate buffer (pH 7.0) to an OD_600_ of 3.0. Then, cells were lysed by sonication and centrifuged at 13,000 × *g* for 12 min. The protein concentration in the supernatant was determined using the BCA Protein Assay Kit (Thermo). LacZ was detected by examining the production of *o*-nitrophenol from ONPG according to a former report [26]. The specific activity of LacZ was calculated according to the corresponding protein concentration and *o*-nitrophenol formation. Production of 1 µmol of *o*-nitrophenol in 1 min by 1 mg total cellular protein means 1 U/mg.

### Detection of the concentration of lysine

Lysine concentrations of the cultures in 96-deep-well plates were detected spectrophotometrically using a specific ninhydrin-ferric reagent that was developed in our lab. Unlike the traditional ninhydrin reaction method which measures the total amino acids, with the specific reagent, the interference of other amino acids could be excluded, and lysine (0–200 mmol/L) in the fermentation broth could be determined accurately and reliably [27]. In this study, the method was further adapted to work with 96-well plates. The specific ninhydrin-ferric reagent was modified to contain 10-g FeCl_3_, 245-mL methylcellosolve, and 3.7-g ninhydrin in 1 L of citric acid-Na_2_HPO_4_ buffer solution (21 g/L of citric acid, adjusted to pH 2.2 with Na_2_HPO_4_). The supernatants (20 μL from each well) of cultures were transferred to new 96-deep-well plates containing 180 μL of the reagent in each well. The plates were sealed and put into a high-pressure steam sterilizer at 105 °C for 40 min. Thereafter, the plates were cooled to room temperature, and 200 μL of dimethyl sulfoxide was added to each well. The reaction mixtures were measured spectrophotometrically at 480 nm. The concentrations of lysine were calculated as previously through a calibration curve obtained with the standard solution of lysine [27]. Lysine concentrations of the cultures in flasks and 5-L fermenters, and glucose concentrations were measured as previously by SBA-40D (Biosensing Analyzer, Shandong, China) [22]. For detection of intracellular lysine concentrations, cells were separated by a silicone oil centrifugation method [28]. The corresponding cell aqueous volumes were calculated according to the former reports [29, 30]. The intracellular lysine concentrations were obtained according to the corresponding intracellular lysine weights and cell aqueous volumes.

### Cell mutagenesis

After overnight cultivation with LB medium, cells were harvested, washed twice, and resuspended in 10 % glycerol to an OD_600_ of 1.0. Mutagenesis was performed with an atmospheric and room temperature plasma (ARTP) mutation system [31]. Cells were treated for 25 s under conditions reported in the previous literature [32].

### Fluorescence analysis and cell sorting by FACS

The cultured cells were washed and resuspended in the potassium phosphate buffer to an OD_600_ of 1.0. Then, cellular EGFP was analyzed with FACS (Beckman Coulter MoFlo XDP) using an excitation line at 488 nm and detecting fluorescence at 529 ± 14 nm at a sample pressure of 60 psi. The diameter of nozzle was set at 70 μm. Sterile filtered phosphate-buffered saline was used as the sheath fluid. Data were analyzed using Beckman Summit 5.2 software.

For selection of the mutant library, a gate containing 0.01 % of the total cells based on the pre-analysis of the mutant library was set to collect the EGFP high-expressing cells. The collected cells were spotted on LB agar plates and further evaluated by fermentation in 96-deep-well plates.

## Results

### Responses of the constructed molecular devices to lysine

**Fig. 1:**
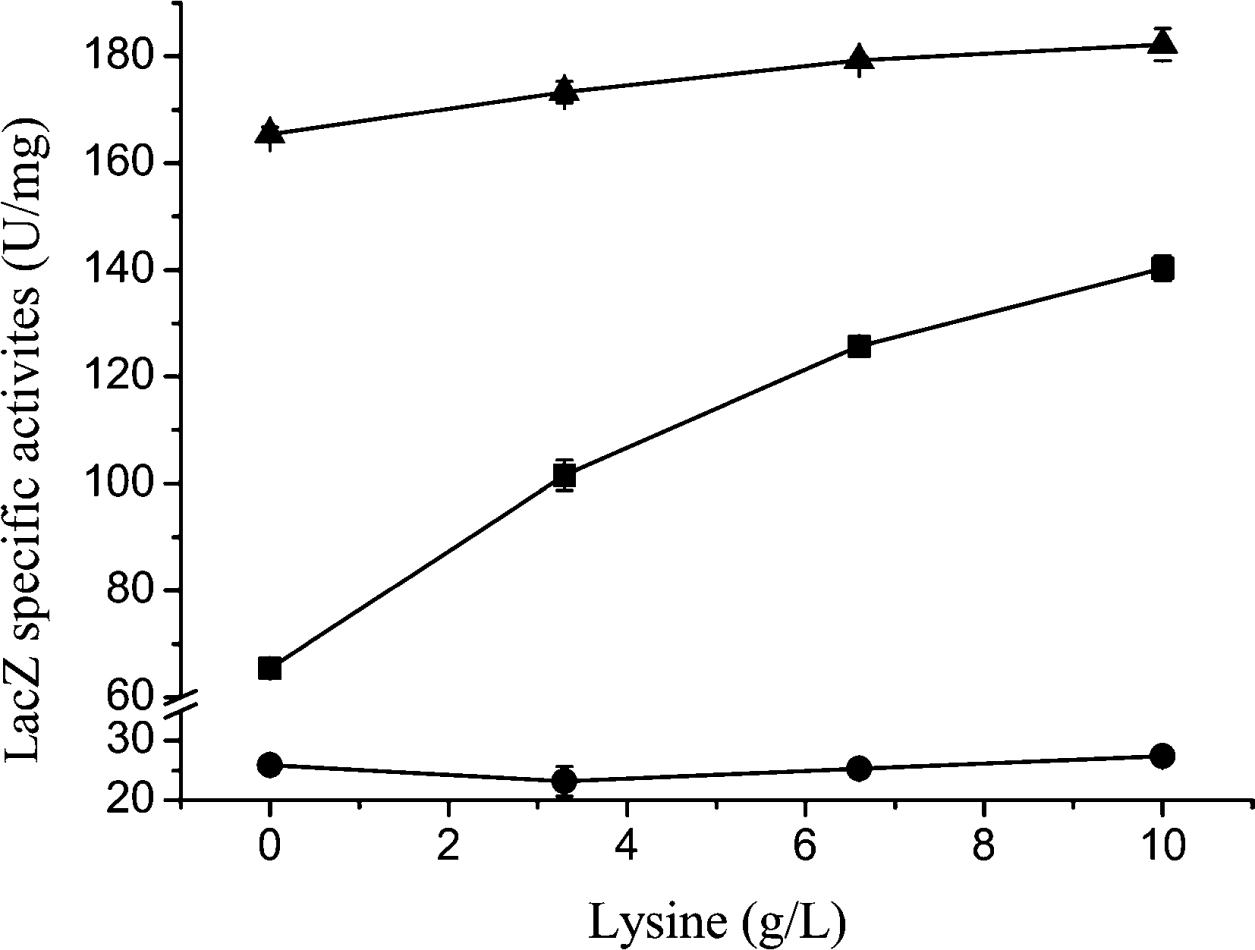
Expression of LacZ in different strains exposed to different concentrations of lysine.* Filled triangle* specific activity of LacZ in LYS2D (pNZ).* Filled square* specific activity of LacZ in LYS2D (pAZ).* Filled circle* specific activity of LacZ in LYS2D (pGZ). Data are shown as the mean and standard deviation of independent triplicates

To determine responses of pN, pA, and lysGE to lysine, plasmids carrying the *lacZ* gene under the control of these devices were constructed and used to transform strain LYS2D. The transformants LYS2D (pNZ), LYS2D (pAZ), and LYS2D (pGZ) were cultured separately for 10 h in LB medium containing lysine at 0, 3.3, 6.6, or 10 g/L. Cultures with 10 g/L of NaCl instead of lysine were used as controls. The LacZ specific activity was detected to deliver quantitative information of the activities of the promoters. As shown in Fig. 1, pN and lysGE in strain LYS2D almost had no response to lysine. The LacZ specific activity of strain LYS2D (pAZ) improved from 65.47 ± 0.11 to 140.29 ± 2.36 U/mg with the increase in lysine concentration from 0 to 10 g/L. The LacZ specific activity of strain LYS2D (pAZ) in the culture with NaCl was 63.59 ± 2.17 U/mg, showing that osmotic pressure was not the factor inducing the activity of pA. These results indicate that the promoter pA is able to respond to lysine added into the culture.

### Establishment of a promoter pA-based biosensor to work with FACS

**Fig. 2:**
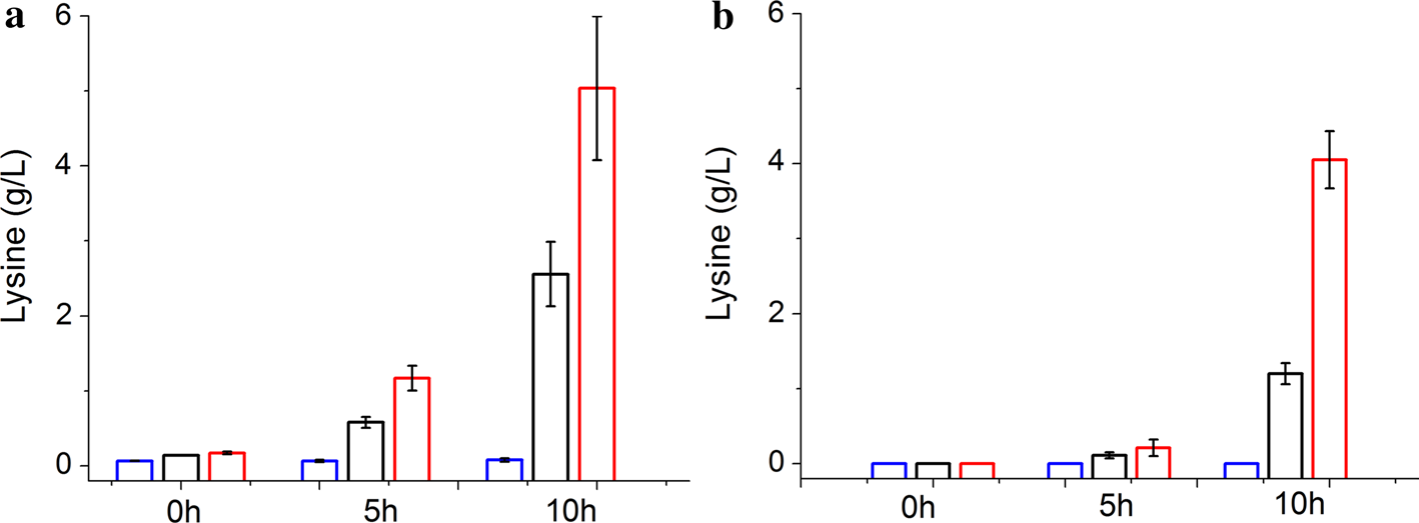
Intracellular and extracellular lysine concentrations of MGl655 (pAG, pTrc99A), LYS1 (pAG), and LYS2 (pAG) at different incubation times.** a** Intracellular lysine concentrations.** b** Extracellular lysine concentrations.* Blue*,* black*, and* red bars* represent data of MGl655 (pAG, pTrc99A), LYS1 (pAG), and LYS2 (pAG), respectively. Data are shown as the mean and standard deviation of independent triplicates

**Fig. 3:**
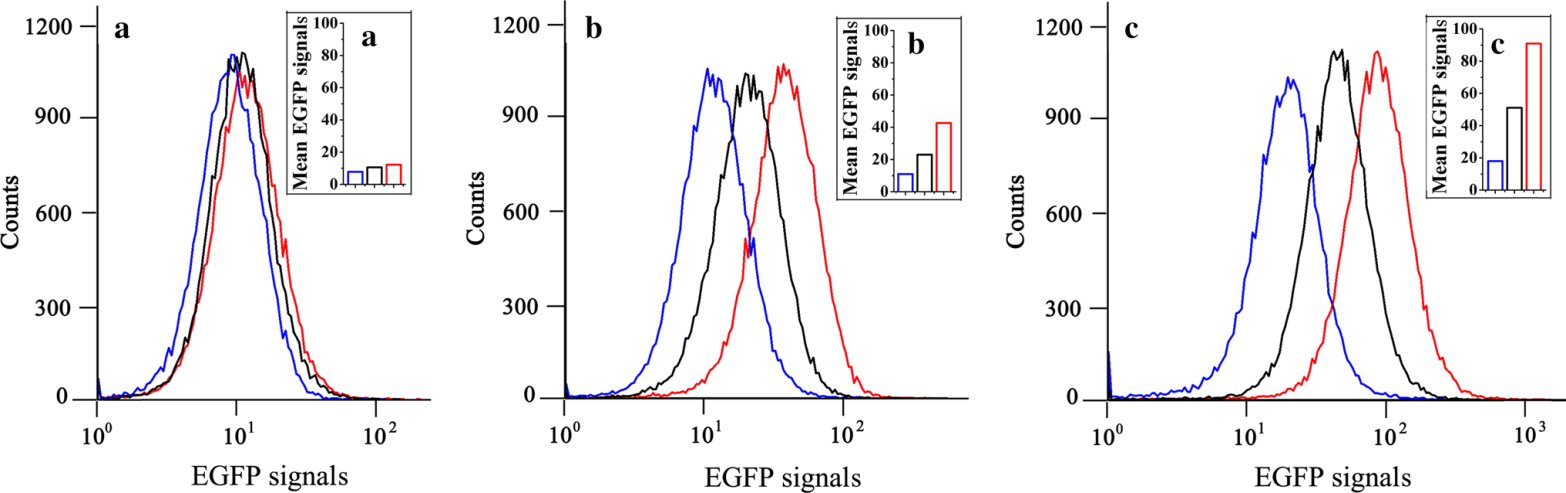
The EGFP expression patterns of MGl655 (pAG, pTrc99A), LYS1 (pAG), and LYS2 (pAG) at different incubation times detected by FACS. A and** a** Incubation for 0 h. B and** b** Incubation for 5 h. C and** c** Incubation for 10 h.* Blue*,* black*, and* red lines* or* bars* represent data of MGl655 (pAG, pTrc99A), LYS1 (pAG), and LYS2 (pAG), respectively

To examine whether pA could respond to lysine produced in vivo, and to establish a biosensor capable of working with FACS, the plasmid pAG carrying an *egfp* gene under the control of pA was constructed. The plasmid pAG was used to transform three strains with different lysine productivities to obtain MG1655 (pAG, pTrc99A), LYS1 (pAG), and LYS2 (pAG). These strains were cultivated in flasks with the fermentation medium. After incubation for 0, 5, and 10 h, the cultures were sampled and analyzed. As shown in Figs. 2 and 3, at 0 h, the intracellular and extracellular lysine concentrations in all of these cultures were very low, and the EGFP expression patterns detected by FACS were difficult to be distinguished. After 5-h incubation, the extracellular lysine concentrations were still very low and no clear difference among different stains (the difference was close to the detection deviations). However, the intracellular lysine concentrations in different cells showed significant variation, from 0.07 ± 0.02 to 1.17 ± 0.17 g/L, and the EGFP expression patterns of different strains could be distinguished significantly from one another. After 10-h incubation, the differences of lysine concentrations and the EGFP expression patterns among the cultures became more obvious. These results indicate that the promoter pA is able to sense endogenous lysine and distinguish *E. coli* strains of different lysine-producing capabilities, and pA-*egfp* has potential to function as a biosensor for developing an FACS-based HTS approach for evolving lysine high-producing *E. coli* strains.

### High-throughput screening of a mutant library and evaluation of the selected mutants

**Fig. 4:**
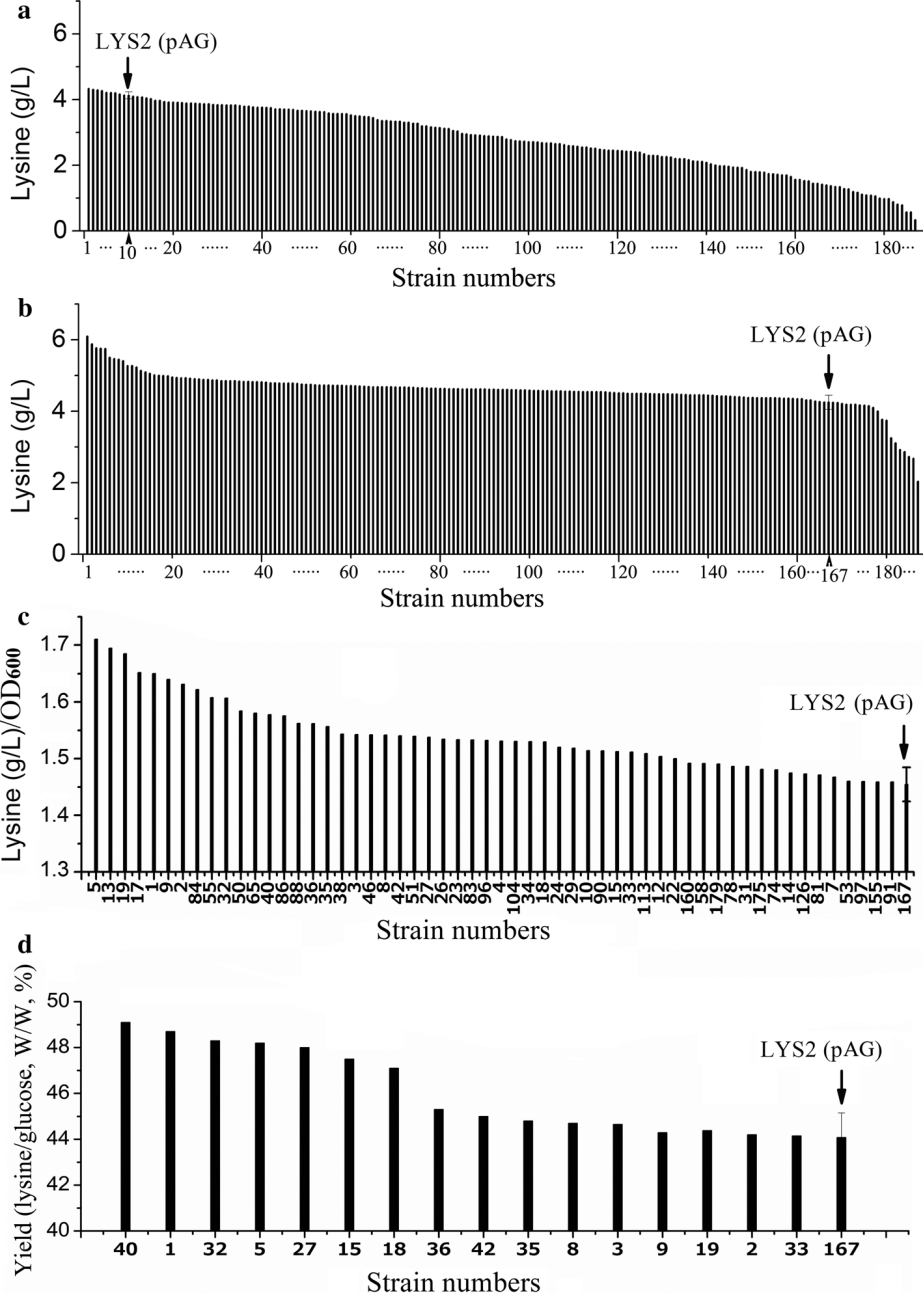
Evaluation of randomly and FACS-selected mutants by fermentation in 96-well blocks.** a** Lysine concentrations of the randomly selected mutant cultures.** b** Lysine concentrations of the FACS-selected mutant cultures.** c** Lysine/OD_600_ ratios of the 56 strains with higher ratios than that of LYS2 (pAG). The 56 strains were selected from the 166 FACS-selected strains with higher lysine concentrations than that of LYS2 (pAG).** d** Lysine yields of the 16 strains with higher yields than that of LYS2 (pAG). The 16 strains were selected from the 56 FACS-selected strains with higher lysine/OD_600_ ratios than that of LYS2 (pAG). The data of the control strain LYS2 (pAG) are shown as the mean and standard deviation of independent triplicates

LYS2 (pAG) cells were treated with the ARTP mutagenesis system. The mutant library was cultivated subsequently for 7 h in a flask containing the fermentation medium to allow accumulation of intracellular lysine and induction of the EGFP expression. Then, 186 cells were obtained by sorting 1 × 10^7^ mutants and subsequent cultivation on LB agar plates (named FACS-selected mutants). In addition, 186 cells were also obtained by collection of the mutants using FACS with a gate containing 100 % of the cells and subsequent cultivation with LB agar plates (named randomly selected mutants). All of the obtained mutants were evaluated by fermentation in 96-deep-well plates. LYS2 (pAG) which served as a control strain was inoculated into three wells in each block. As shown in Fig. 4a, b, after cultivation for 16 h, 166 of the 186 FACS-selected mutants produced lysine with higher concentrations than that of LYS2 (pAG), and 72 with concentrations improved by more than 10 %. None of the randomly selected mutants produced lysine with a concentration improved by more than 5 %. These results confirmed that FACS with the aid of the pA-*egfp*-based lysine biosensor could highly elevate the positive rate of mutant screening. Lysine/OD_600_ ratios of the 166 strains were analyzed, and 56 strains have higher ratios than that of LYS2 (pAG). The lysine/OD_600_ ratios of LYS2 (pAG) and these 56 strains are shown in Fig. 4c. The yields (lysine/glucose, W/W) of the 56 strains were further analyzed, and 16 strains have higher yields than that of LYS2 (pAG). The yields of LYS2 (pAG) and these 16 strains are shown in Fig. 4d. When evaluating the FACS-selected strains according to the lysine concentration, the lysine/OD_600_ ratio and the yield of lysine from glucose, number 1 and 5 mutants were always of the top 10 (Fig. 4b–d). They were named MU-1 and MU-2, and selected for further analyses.

### Fed-batch fermentations of the mutants MU-1 and MU-2, and the original strain LYS2 (pAG)

**Fig. 5:**
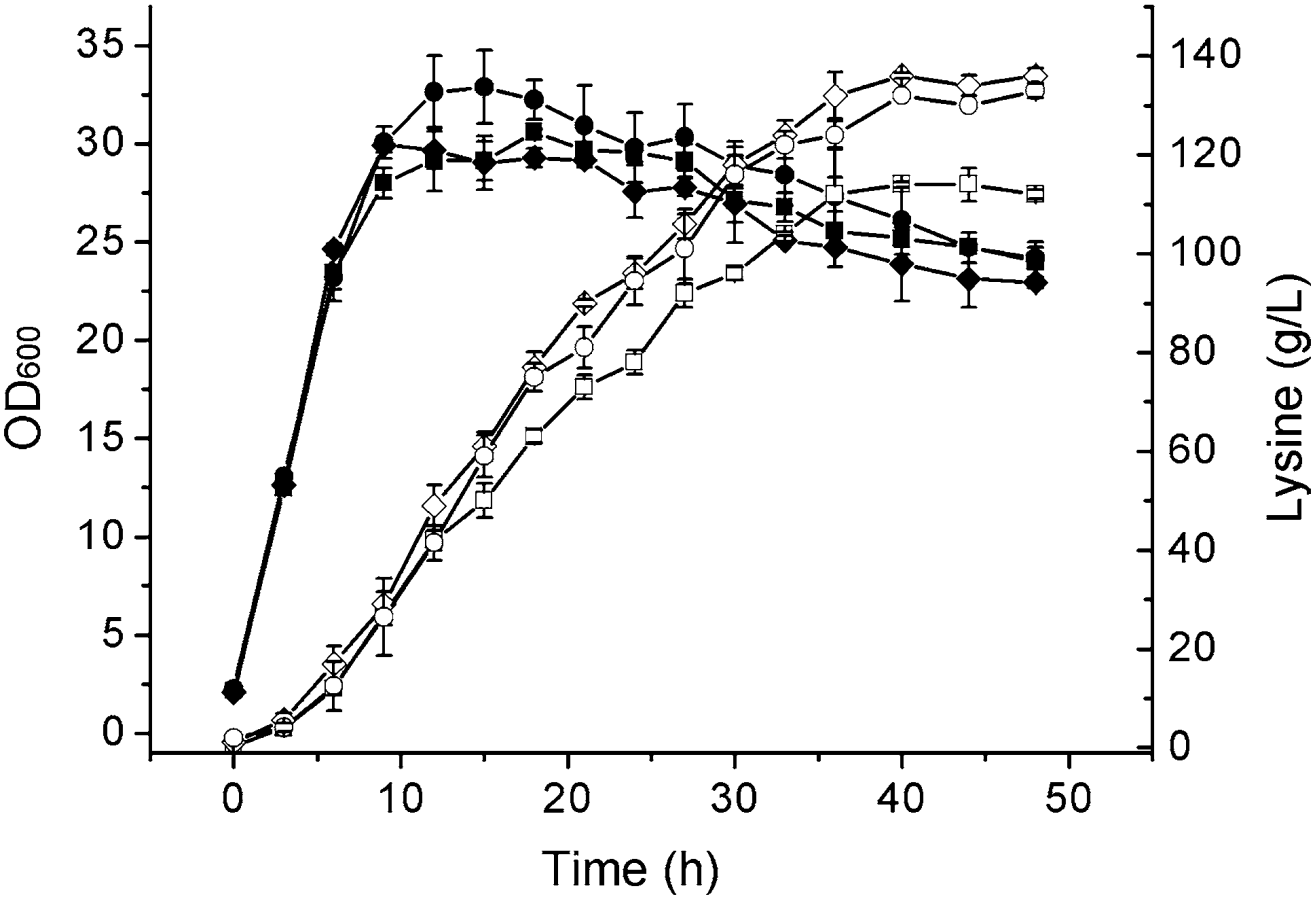
Cell growth curves and lysine production of MU-1, MU-2, and LYS2 (pAG) during the fed-batch fermentations.* Filled diamond* OD_600_ of MU-1 cultures.* Filled circle* OD_600_ of MU-2 cultures.* Filled square* OD_600_ of LYS2 (pAG) cultures.* Unfilled diamond* lysine concentrations of MU-1 cultures.* Unfilled circle* lysine concentrations of MU-2 cultures.* Unfilled square* lysine concentrations of LYS2(pAG) cultures. Data are shown as the mean and standard deviation of independent triplicates

The lysine-producing capacities of mutant strains MU-1 and MU-2 were further tested in 5-L jar fermenters. LYS2 (pAG) was used as a control strain. Fermentations were continued for 48 h. Cell growth and lysine production are shown in Fig. 5. Lysine concentrations, productivities, and yields at the end point of fermentations are shown in Table 2. Compared with the lysine concentration, the productivity, and the yield of the original strain LYS2 (pAG), those of strain MU-1 improved by 21.00, 20.85, and 9.05 %, respectively, and those of strain MU-2 improved by 18.14, 18.30, and 10.41 %, respectively.

**Table 2:** Comparison of lysine production of the selected mutants with the parent strain

Strains	Lysine concentrations (g/L)	Productivities g/(L h)	Yields (lysine/glucose, g/g, %)
LYS2 (pAG)	112.82 ± 0.97	2.35 ± 0.02	50.83 ± 1.25
MU-1	136.51 ± 1.55	2.84 ± 0.03	55.43 ± 0.47
MU-2	133.29 ± 1.42	2.78 ± 0.03	56.12 ± 1.37

## Discussion

Strains of *E. coli* are important industrially using lysine producers. Because of the fact that it is difficult to further improve the industrial strains relying on pure rational design [33], a knowledge-less-dependent HTS method is desired. However, such a method is not available to work with the lysine high-producing *E. coli* strain up to now. The devices lysGE, pN, and pA from *C. glutamicum* 13032 were reported to be capable of responding to lysine from zero up to several grams per liter [20, 21]. A lysGE-based biosensor was constructed for developing a HTS method to evolve lysine-producing *C. glutamicum* strains [21]. Different strains, even those with close evolutionary relations in phylogenetic analyses, might harbor different but important genetic variations and regulations, which might affect the function of a biosensor. It is preferable to use an industrial strain to test the response of the biosensor if the objective is clearly set to improve the production of this strain. Although lysGE, pN, and pA in *C. glutamicum* strains could respond to lysine, whether they could respond to lysine in *E. coli* strains was unknown. In this study, we evaluated responses of these devices to lysine in the lysine non-producing *E. coli* strain LYS2D that was derived from the lysine high-producing strain LYS2 by elimination of its plasmid pTrc99A-*dhdps*-aspk [22]. The results shown in Fig. 1 proved that the promoter pA but not pN or lysGE should be useful for constructing a biosensor working in E. coli strains.

To distinguish different mutant cells with various lysine productivities, an ideal biosensor should respond to diverse endogenous lysine concentrations. Strain LYS2 is a fine lysine producer (Fig. 5). It was derived from LYS1. As shown in Fig. 2a, after incubation for 10 h in flasks under the uncontrolled fermentation condition, the intracellular lysine concentration of LYS2 was 5.04 ± 0.96 g/L, about 1.97 and 62.99 times those of LYS1 and MG1655, respectively. When EGFP was used as a signal to work with FACS, LYS2 (pAG), LYS1 (pAG), and MGl655 (pAG, pTrc99A) could be distinguished obviously (Fig. 3). The regulatory mechanism for the response of pA to lysine was unknown, and the potential regulatory element was not concerned in our study. It was reported that a chemical-inducible promoter and a reporter gene were enough to be used for constructing an efficient biosensor for high-throughput selection of mutant strains producing the target chemical [26]. Our subsequent experiments also proved that the pA-based biosensor was efficient enough for high-throughput selection of LYS2 mutants.

Figure 1 shows that pA can respond to the change of extracellular lysine concentration. It could be speculated that lysine excreted by all strains in the mutant library might affect the intracellular EGFP level of an individual cell. As a result, it might affect the proper screening of desired mutants by FACS. Therefore, the mutant library should be screened before the extracellular lysine concentration had risen up to a significant level in the culture. As shown in Figs. 2 and 3, after cultivation for 5 h, the tested strains with different intracellular lysine concentrations were distinguished obviously by FACS. Meanwhile, the extracellular lysine concentrations were in relatively low levels. Therefore, a 5-h preculture stage should be suitable for preparing the mutant cells for FACS selection. Considering the cells should be injured by the treatment of the ARTP system, the preculture was prolonged for two more hours, namely, the mutant library was screened by FACS after a preculture of 7 h. As shown in Fig. 4a, b, with the lysine concentration as a parameter for evaluation of the selected mutants, up to 89 % of the FACS-selected strains were better than the original strain, much more than that of the randomly selected strains, proving the selection procedure to be appropriate.

The concentration and the yield of lysine are mostly concerned parameters affecting the economic and environmental performance of the industrial production. The concentration of lysine is determined mostly by cell density and productivity per unit of cell. The cell density is adjustable to a certain extent in controllable industrial fermenters. In 96-well blocks where the cell density was not easily controlled, productivity per unit of cell should also be an important parameter for evaluating the activities of mutants. In our study, the concentration and the yield of lysine, the productivity per unit of cell, were all considered as parameters for evaluation of the FACS-selected mutants (Fig. 4a–d), and MU-1 and MU-2 that were always of the top ten mutants were selected. Further experiments using 5-L fermenters proved that the lysine-producing capabilities of MU-1 and MU-2 improved a lot. Compared with the traditional mutant evaluation methods using product concentration as the only parameter, our evaluation based on various parameters should be more accurate. The lysine concentrations, productivities, and yields of both of MU-1 and MU-2 are all higher than those of the previously reported best lysine-producing E. coli strain [10].

It took about only 2 weeks to complete one round of strain HTS and further evaluation process for a 1 × 10^7^-mutant library, which is 10^4^–10^5^ times faster than the traditional shaking flask culture-based mutant library selection methods. The results indicate that the strain selection and evaluation system is highly efficient and time-saving. The system established in our study should be useful for continuous improvement of the current E. coli strains in the lysine industry.
